# Supplementary material for: Fast and versatile electrostatic disc microprinting for piezoelectric elements
Source: Nat Commun. 2023 Oct 14;14:6488. doi: 10.1038/s41467-023-42159-9 (PMC10576804; doi:10.1038/s41467-023-42159-9)
Supplement: Supplementary file 1 — Supplementary Information [file 41467_2023_42159_MOESM1_ESM.pdf]

Supplementary Information for

Fast and Versatile Electrostatic Disc Microprinting for Piezoelectric Elements

Xuemu Li[1,2,3], Zhuomin Zhang[1,2,3], Zehua Peng[1,2], Xiaodong Yan[1,2], Ying Hong[1,2], Shiyuan Liu[1,2], Weikang Lin[1,2], Yao Shan[1,2], Yuanyi Wang[1], Zhengbao Yang[1,2]*

1. Department of Mechanical and Aerospace Engineering, Hong Kong University of Science and Technology, Clear Water Bay, Hong Kong, China
2. Department of Mechanical Engineering, City University of Hong Kong, Hong Kong, China
3. These authors contributed equally to this work: Xuemu Li, Zhuomin Zhang.

* Correspondence to: Zhengbao Yang (zbyang@ust.hk).

This PDF file includes:

Supplementary Notes 1 to 3
Supplementary Figures 1 to 22
Supplementary Tables 1 to 6
Supplementary References

Supplementary Notes

S1. Formation of multiple jets

By means of conformal mapping of a thin ellipsoid, the electric field around the thin disc can be expressed as follows,

$$E = \frac{CV}{\sqrt{D\delta}} \tag{S1}$$

where C is a geometry correction factor of order 1, V is the applied voltage, δ is the combined thickness of liquid and disc near the edge of the disc, and D is the distance between the disc and substrate. The minimum electric field E_0 is needed to enable electric stress overcoming the surface tension: $\sigma/\delta \sim \varepsilon_0 E_0/2$ (σ is the surface tension coefficient), so the onset voltage is:

$$V_0 = \sqrt{2\sigma H / \varepsilon_0 C^2} \tag{S2}$$

A thin disc can achieve multi-jetting because of its special geometric features: 1) the thin disc with extremely small thickness and the tips evenly distributed around the edge of disc contribute to the very intense electric field; 2) the nonsmall spacing between the protruding tips effectively avoids the mutual interference between the jets.

Actually multi-jet mode may be formed from a capillary with single bore, in which these cones often move and even spin around the axis of the capillary. To overcome such rather unstable mode, higher electrical field or sharp features added at the outlet of the capillary are usually applied to preferably anchor these jets. In contrast, the EDP process from a thin spiny disc is very stable. One reason may be that the liquid film is thin and the viscous effect is sufficient to damp against perturbations. For a single Taylor cone of liquid from the capillary, back-flow usually appear inside the tapering liquid meniscus, which will jeopardize the cone. The thin disc, serving as a planar insert, can help to break the back-flow and improve the stability of liquid flow.

The dimensions of the thin spiny disc used in our work is shown in Fig. S1a. Its diameter of addendum circle and dedendum circle is 5.5 mm and 5 mm, respectively. The topology of the multi-tips design helps trigger liquid-air interface instability at the rim of the disc, which is critical to generating multiple radial liquid ligaments.

By applying positive potential to different wetted spiny discs (spiny disc design in Fig.S1), multiple liquid jets are issued from the tips of disc, forming a symmetric radial jet mode (Fig. S2 and Fig. S3). As the increase of the diameter of disc (D, from 3.5 mm to 10.5 mm), the applied voltage for generating stable cone-jets raise. For the small discs (D=3.5 mm), the ink is easy to flow out of the disc and drop onto the substrate, which inevitably will disrupt the uniformity of the deposited film. The liquid jet undergo Rayleigh-Plateau instability and will be split into droplet clusters with a diameter twice that of the jet. The amount of atomized droplets can be fine-tuned by controlling the number of tips (N). We find a well-defined optimum in atomization stability and productivity around N=16. At small N, atomizing yield is inhibited by the decreasing role of tip streaming; at high N, the mutual interference of jets/droplets is stronger, which affects the stability of jets.

S2. Particle size depending on sol concentration

The relationship between sol viscosity and atomized droplet size is based on a dimensionless parameter π_η [1],

$$\pi_\eta = \frac{\sqrt[3]{\gamma^2 \rho \varepsilon \varepsilon_0}}{\eta} \tag{S3}$$

where γ is the surface tension, ρ is the density, ε is the relative permittivity, ε_0 is the vacuum permittivity and η is the viscosity. If π_η <<1, the droplet size will increase with the viscosity, so the increasing sol concentration can result in the increase of droplet size and hence in particle size. During depositing of PZT ink, the relationship between sol properties and droplet diameter can be described by the following equation[2],

$$d \propto \left(\frac{Q^3 \varepsilon_0 \rho}{\gamma K} \right)^{1/6} \tag{S4}$$

where Q and K is the flow rate and electrical conductivity of the PZT sol. Another equation, valid when $\varepsilon >> 1$, is given by the following equation[3],

$$d \propto \varepsilon^{1/6} \left(\frac{Q \varepsilon_0}{K} \right)^{1/3} \tag{S5}$$

These two equations provide ways to predict the droplet size based on the properties of the sol. Shrinkage is inevitable in the sol system and should be taken into account when predicting particle size.

Here, two modified equations are obtained by assuming the volumetric shrinkage term (cube root of the sol concentration). Both the predicted results and the observed results are shown in Fig. 3g. It can be seen that the observed trend of particle diameter as a function of the sol concentration is in agreement with the predicted results. The particle diameter increases with increasing sol concentration. However, the discrepancy between the observed value and the predicted value becomes noticeable with the increase of sol concentration. This mismatch may be attributed to the viscosity effect, which is generated by the concentration gradient and the evaporation rate difference.

S3. Leakage current characteristics of EDP PZT films

Normally, the long-time service process of the piezoelectric films is accompanied with the increase of the leakage current, eventually resulting in the breakdown. The above phenomenon is called as the electric degradation. Here, the leakage current measurements of our PZT films with Ag electrodes is conducted under a DC bias field of 150 kV/cm and a temperatures of 180 °C. The applied electric field is directed from the top to the bottom electrode. Ag top electrodes with diameters of 1 mm are printed onto the PZT surface by EDP (Fig. S22a). Measurements of current are made 60 s after any change in value to allow the current to stabilize. Fig. S22b presents the characteristic leakage current response from our PZT film. The steady-state current rises gradually upon resistance degradation.

Supplementary Figures

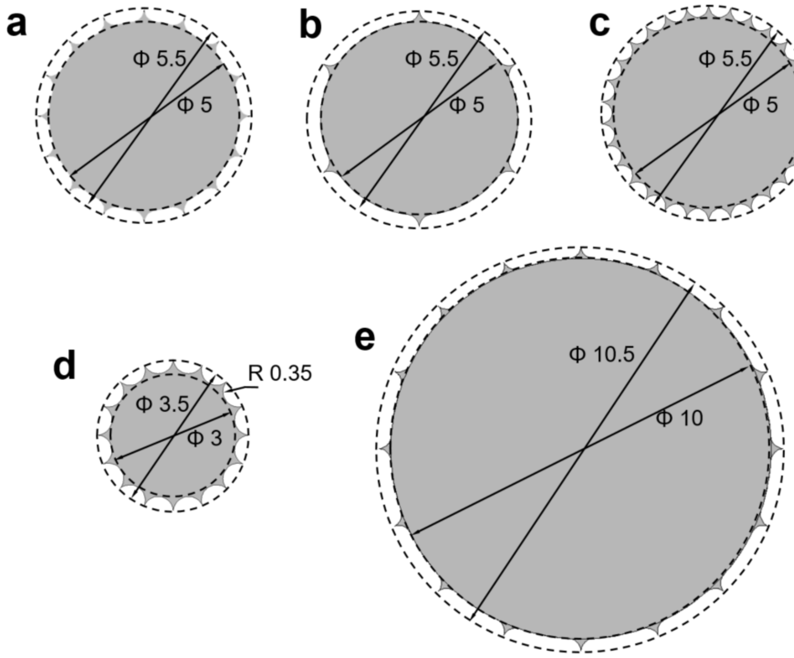

Fig. S1. Dimensions of thin spiny discs. Disc with maximum radius of 5.5 mm and 16 tips (a), disc with maximum radius of 5.5 mm and 6 tips (b), disc with maximum radius of 5.5 mm and 30 tips (c), disc with maximum radius of 3.5 mm and 16 tips (d), and disc with maximum radius of 10.5 mm and 16 tips (e).

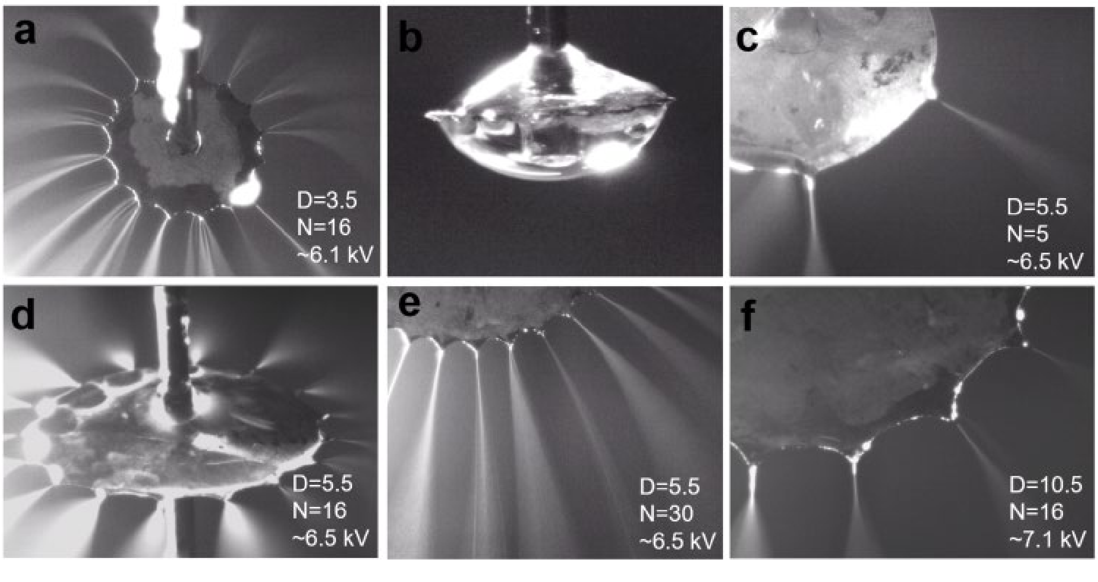

Fig. S2. Multiplexed EDP tip jetting from the thin disc with diameter (D) of 3.5 mm and tips number (N) of 16 (a). Droplets flowing out of the disc (D=3.5, N=16) (b). Multiplexed EDP tip jetting from the thin disc (D=5.5 mm, N= 5 (c). Multiplexed EDP tip jetting from the thin disc (D=5.5 mm, N= 16) (d). Multiplexed EDP tip jetting from the thin disc (D=5.5 mm, N= 30) (e). Multiplexed EDP tip jetting from the thin disc (D=10.5 mm, N= 16) (f). The applied voltage for generating stable cone-jets raise as the increase of the disc diameter. Liquid: ethanol; supply rate of ink: 10 μl min^{-1}; distance between disc and substrate: ~5 mm.

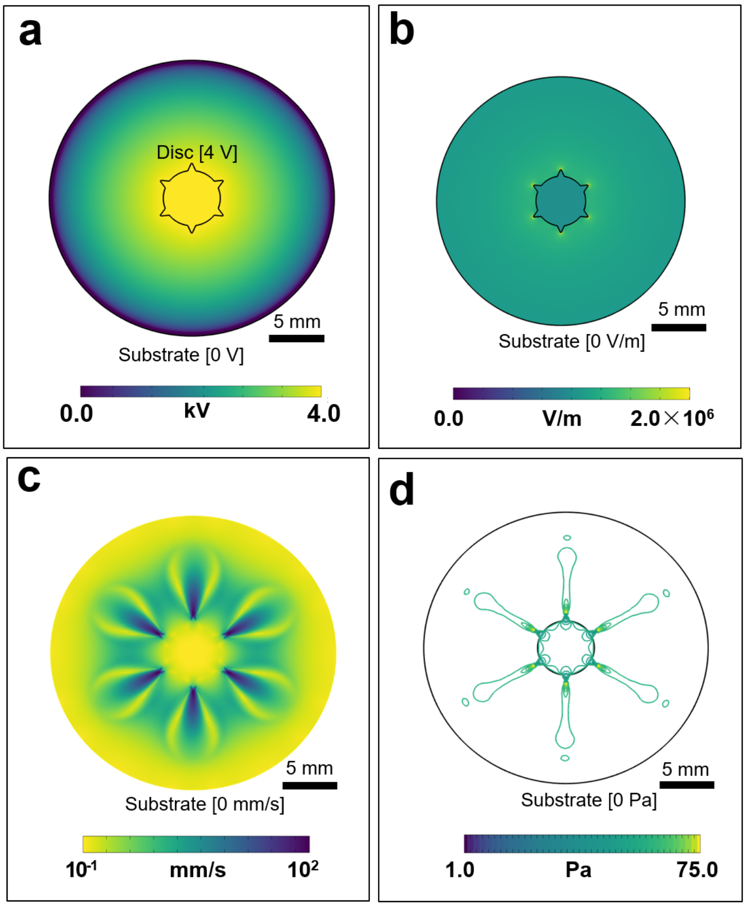

Fig. S3. Finite element analysis. Simulation of the electric potential (a), electric field (b), fluid velocity (c) and fluid pressure (d) around the disc (with six tips) during EDP.

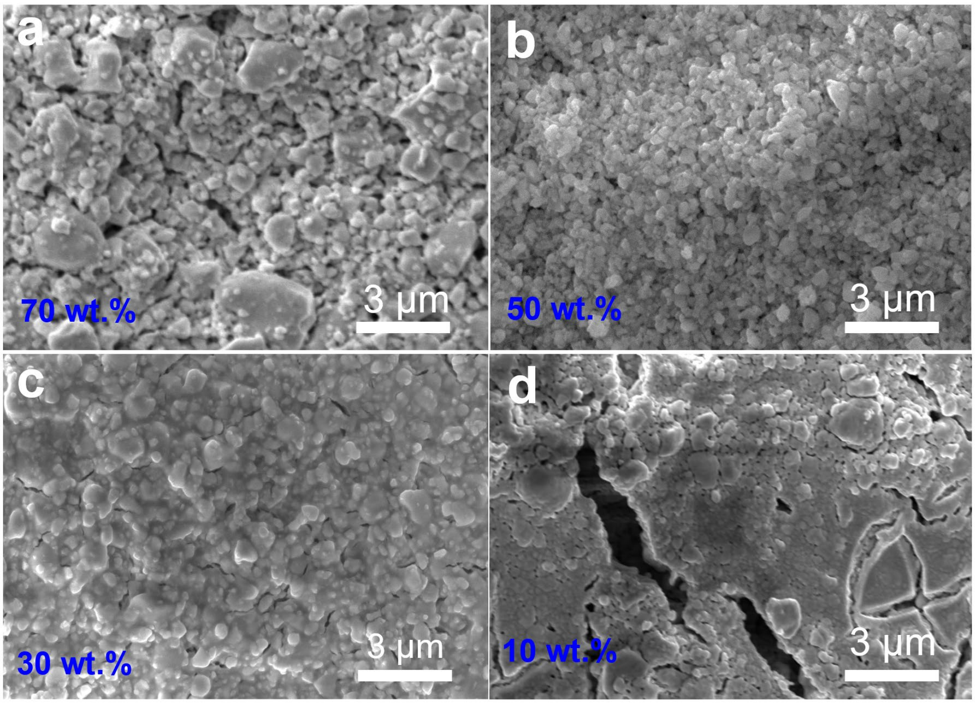

Fig. S4. SEM images of surface topography of the deposited PZT films using PZT ink with different concentrations: 70 wt.% (a); 50 wt.% (b); 30 wt.% (c); 10 wt.% (d). The films deposited using the 70 wt.% PZT slurry show porous feature. With the decrease of slurry concentrations (50-30 wt.%), the porosity of films decreases. When the slurry concentration is further decreased, the films show distinct cracks due to the high level of shrinkage induced stress in the sol during drying and pyrolysis. The viscosity of PZT slurry with different concentrations (70 wt.%, 50 wt.%, 30 wt.%, and 10 wt.%) is ~1.6, ~1.4, ~1.1, and ~1.0 mPa s. The applied voltages for stable EDP process using different inks (concentrations of 70 wt.%, 50 wt.%, 30 wt.%, and 10 wt.%) are ~3 to ~10 kV.

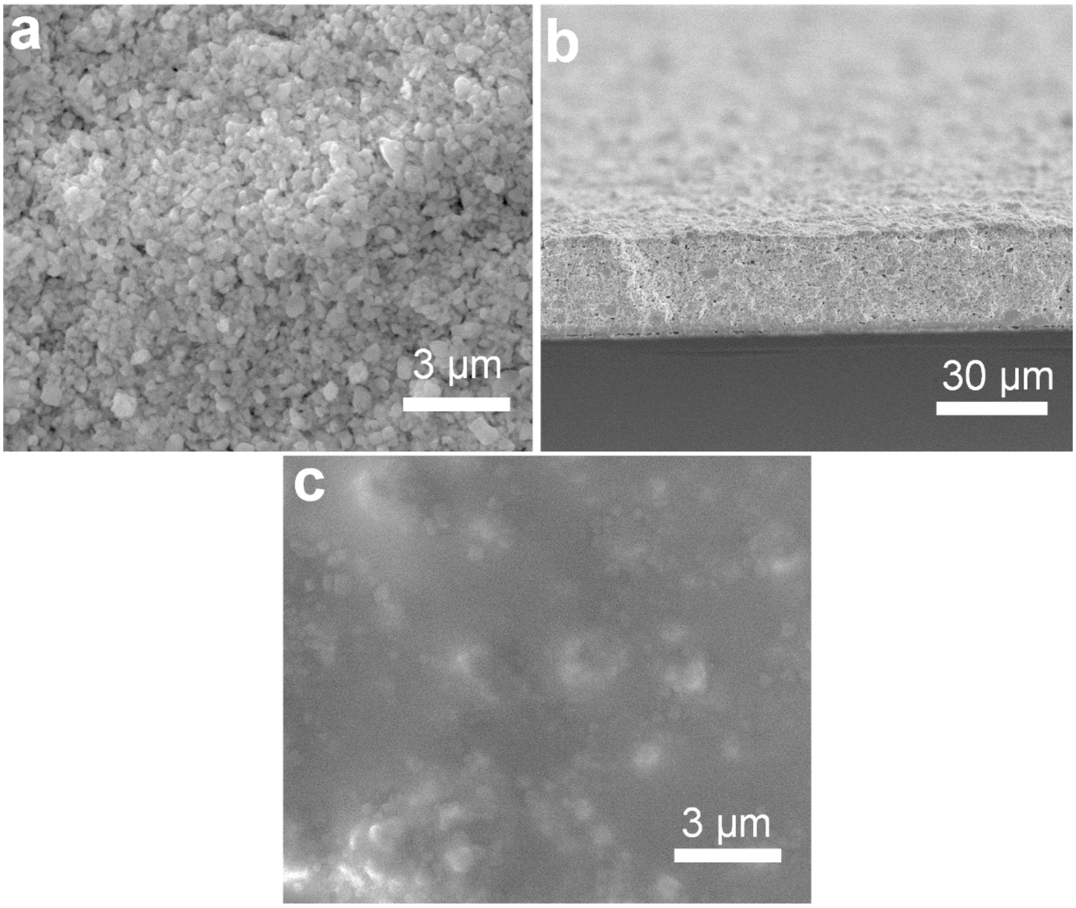

Fig. S5. SEM images of surface topography (a) and cross-sectional topography (b) of the as-deposited PZT films. Surface topography of the sol-infiltrated PZT films (c).

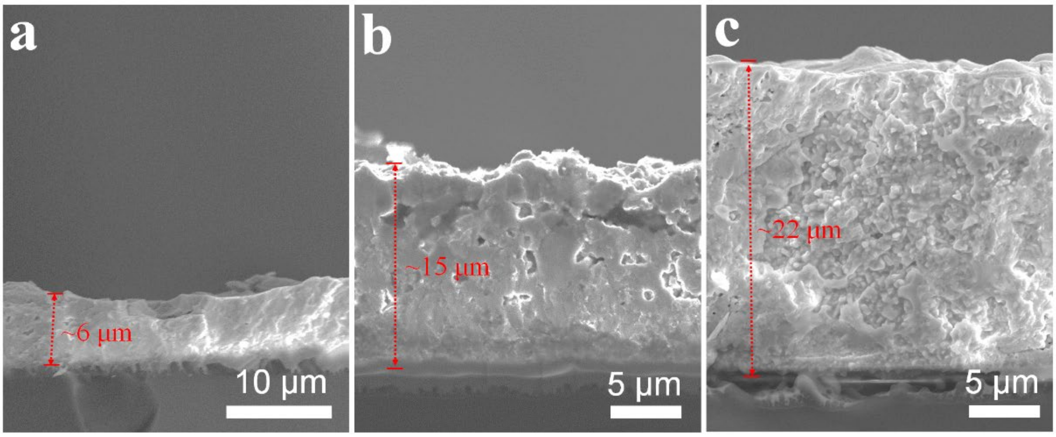

Fig. S6. The cross-sectional topographies of annealed PZT films with different thickness.

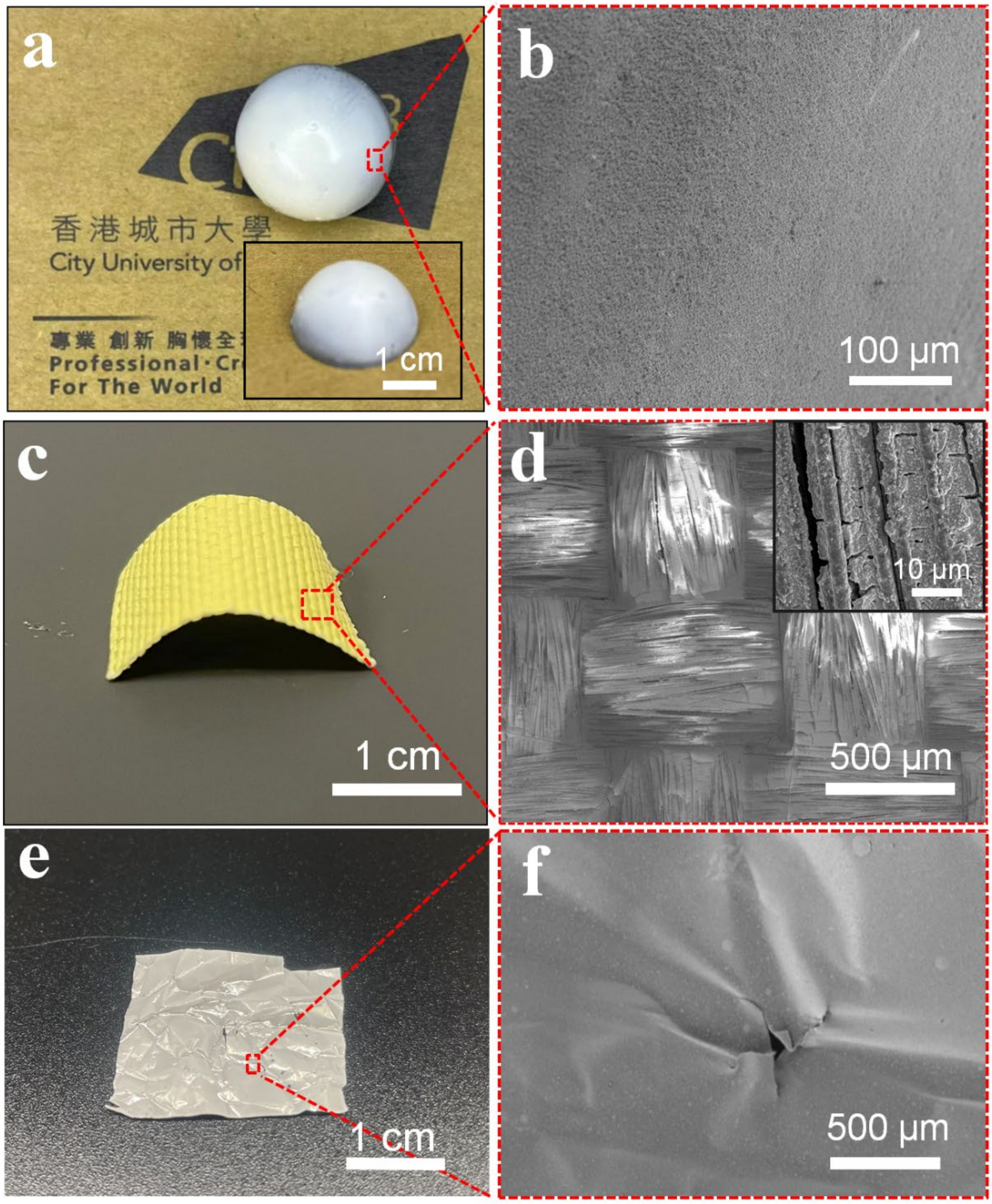

Fig. S7. Optical photographs of PZT films deposited on spherical surface (a) and the corresponding SEM image (b). Optical photograph of PZT films deposited on cloth substrate (c) and the corresponding SEM images (d). Optical photograph of PZT films deposited on wrinkled steel sheet (e) and the corresponding SEM images (f).

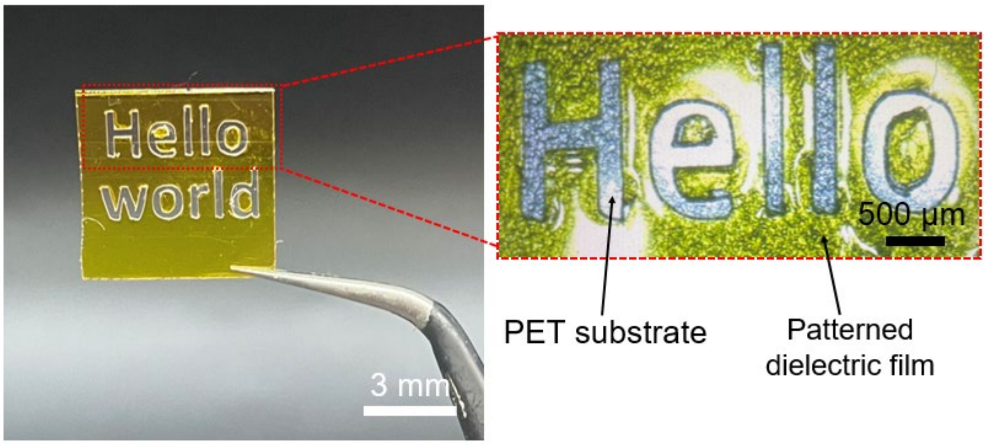

Fig. S8. Optical photographs of polyethylene terephthalate (PET) substrates (transparent area) covered with polyimide masks (yellow area).

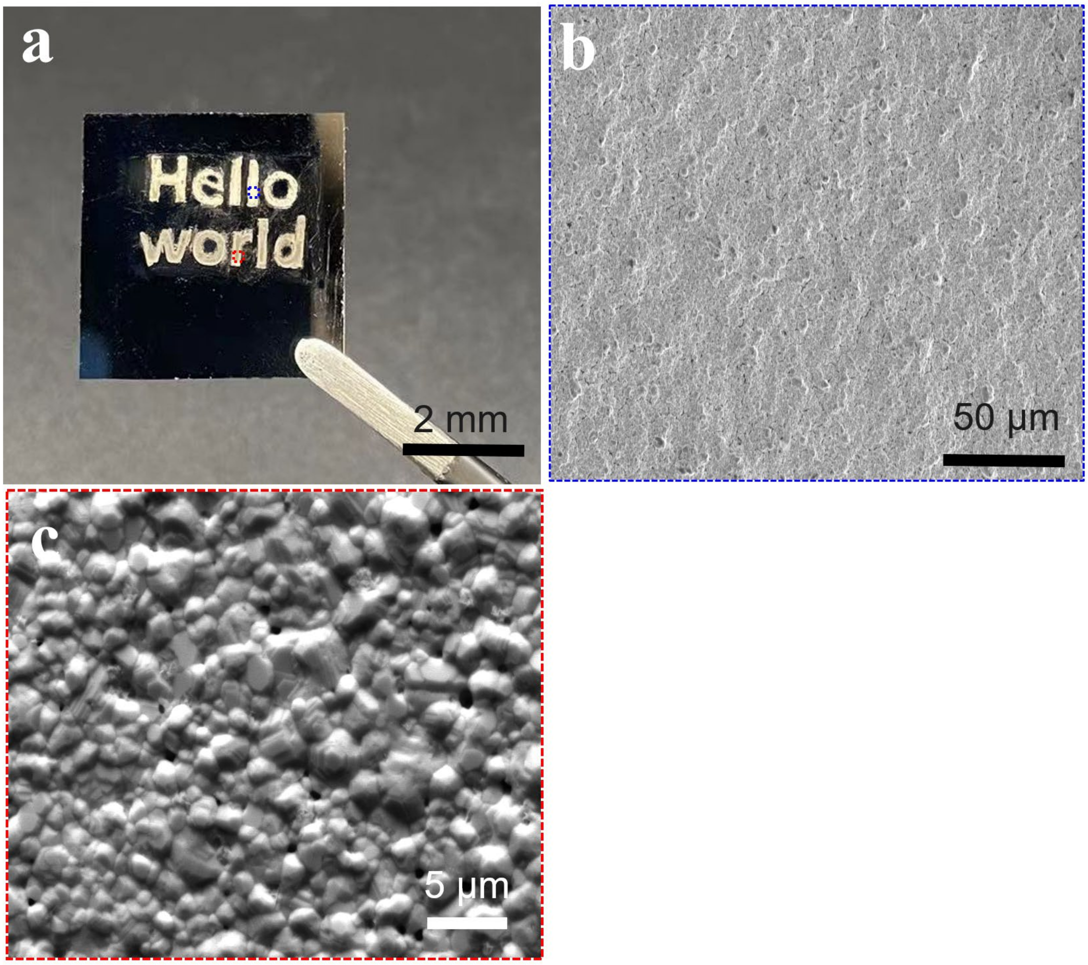

Fig. S9. Optical photograph of printed letters ("Hello world") on the Si substrate (a) and the magnified SEM image of PZT part (b) and Ag part (c).

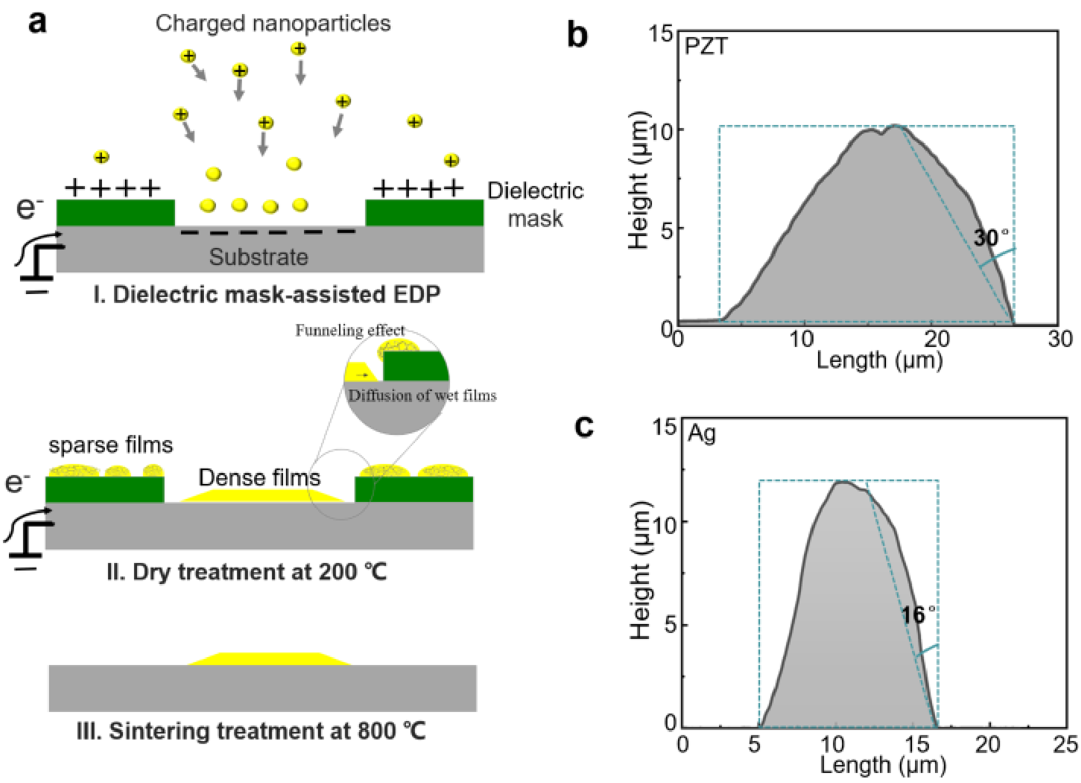

Fig. S10. The processing steps to fabricate micropatterns (a). Line profile taken from the AFM image of the PZT surface shown in Fig.3d (b). Line profile taken from the AFM image of the Ag surface shown in Fig.3d (c).

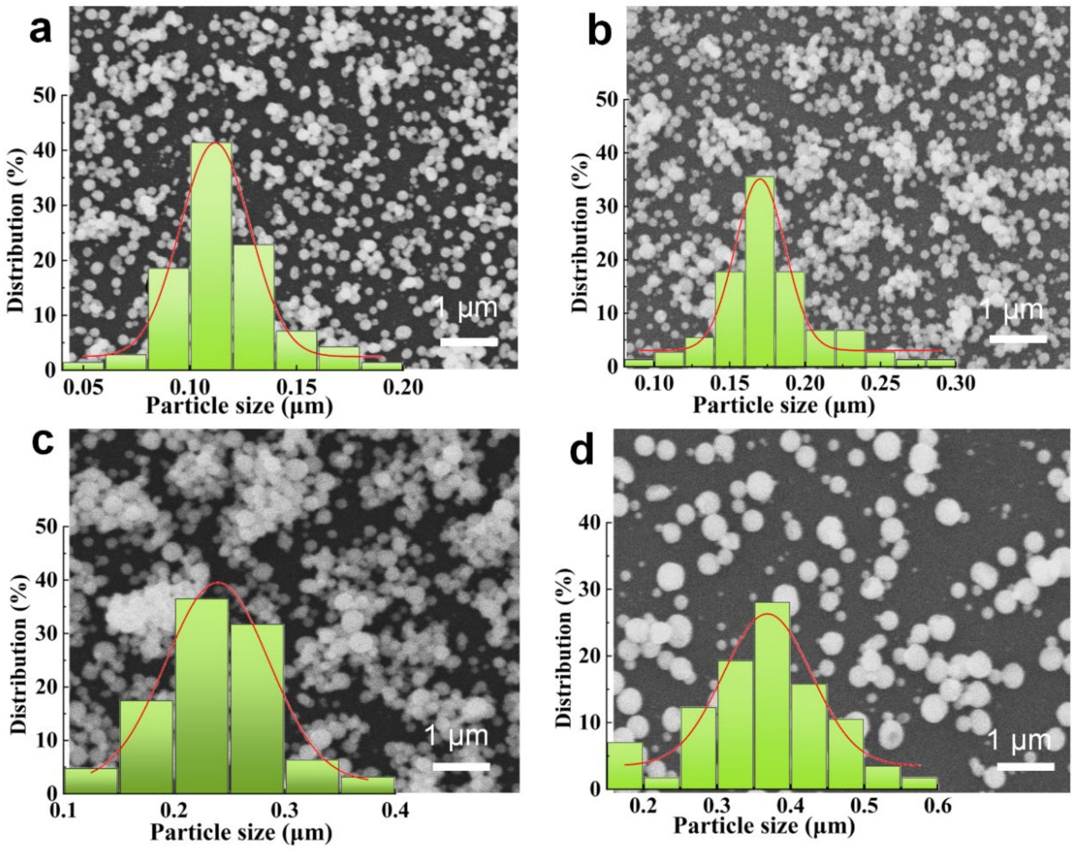

Fig. S11. SEM micrographs of PZT particles obtained from different sol concentrations and the corresponding particle size distribution curves: (a) 0.1 M; (b) 0.2 M; (c) 0.3 M; (d) 0.4 M.

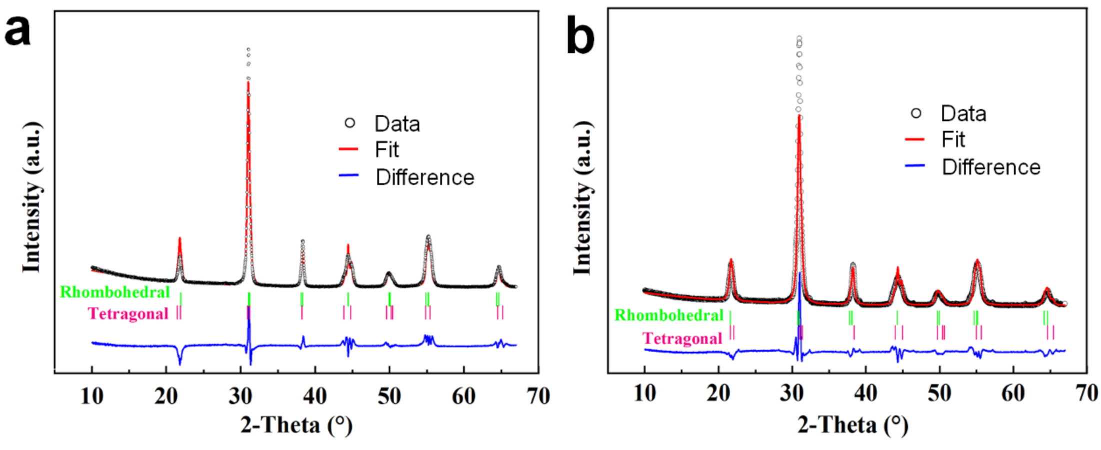

Fig. S12. Measured and modeled X-ray diffraction patterns for the PZT films fabricated by dip-coating (a) and EDP (b).

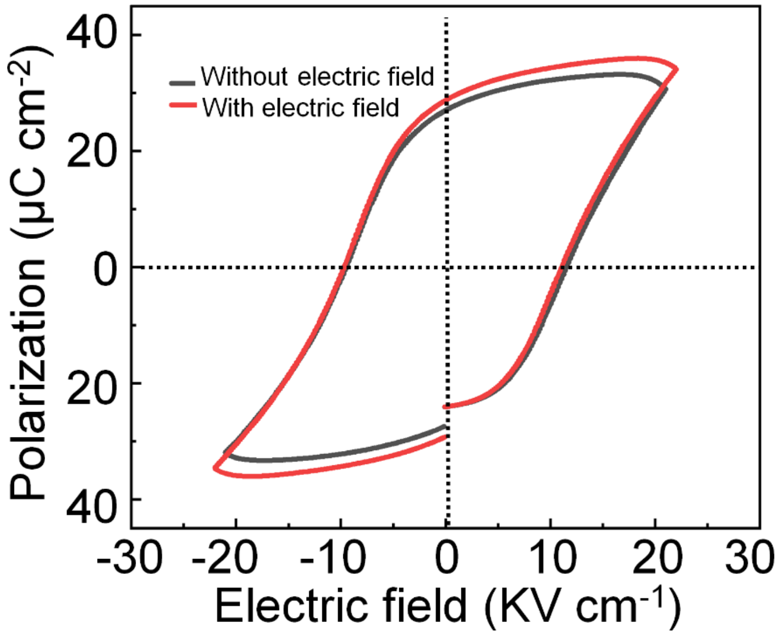

Fig. S13. The polarization-electric field (P-E) ferroelectric hysteresis loops of the annealed PZT films fabricated via EDP and dip-coating at room temperature (RT) and at 10 Hz.

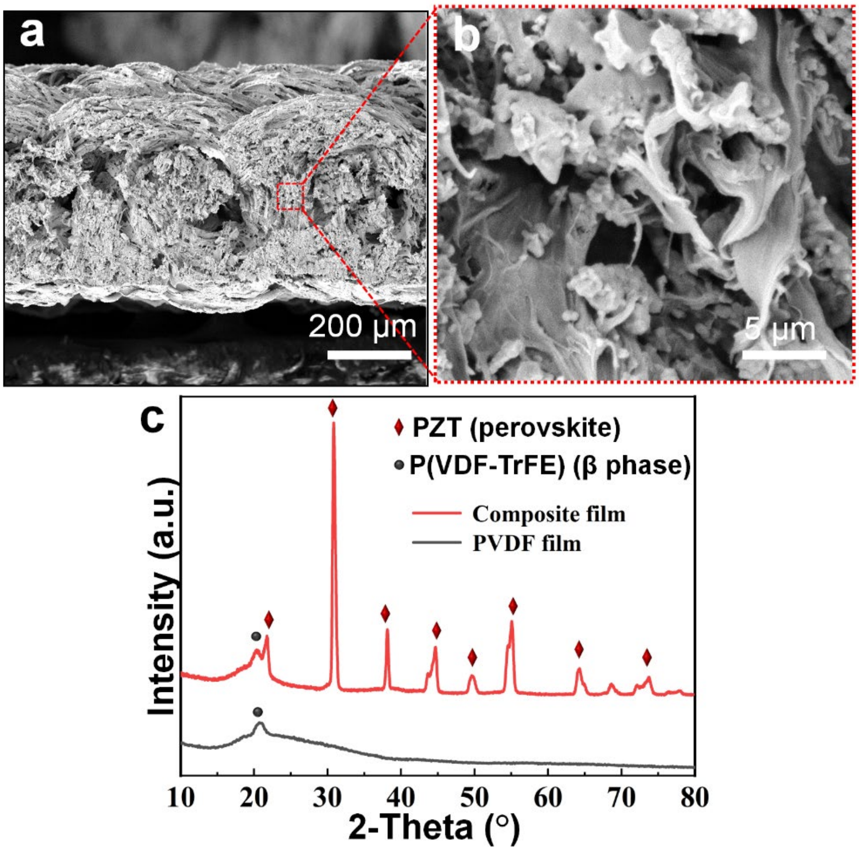

Fig. S14. The SEM cross-sectional image of the piezocomposite framework showing the thickness of ~500 μm (a) and PZT fibers coated with P(VDF-TrFE) (b). X-ray diffraction patterns of PZT/P(VDF-TrFE) piezocomposite framework and pure P(VDF-TrFE) film (c), showing the typical characteristic peaks of perovskite structure and β phase.

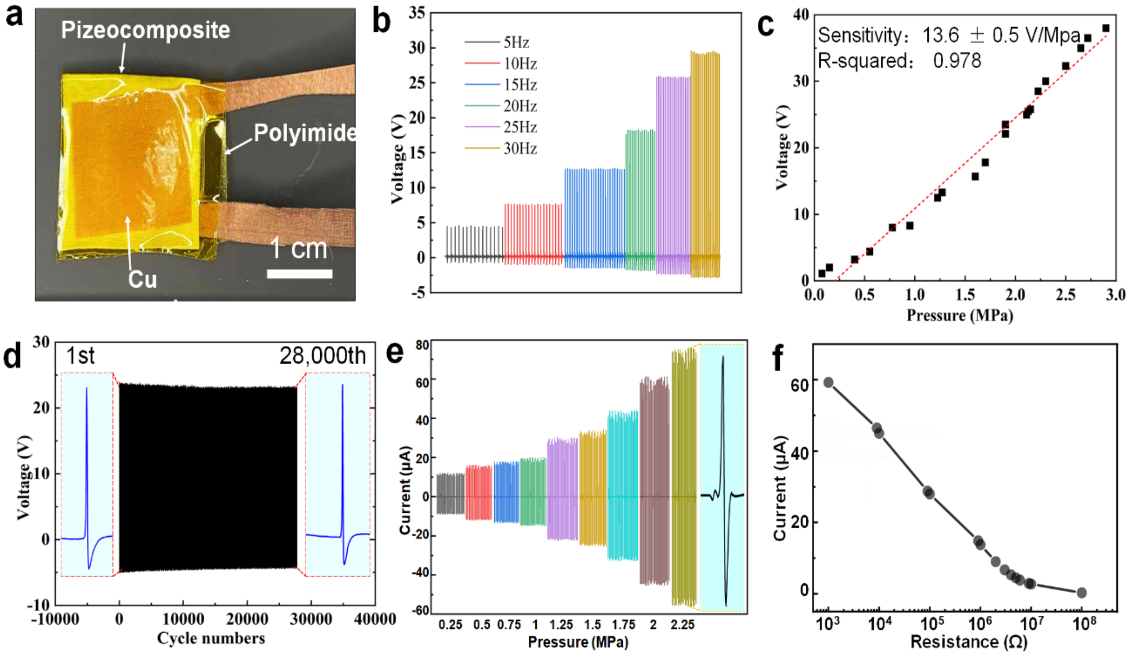

Fig. S15. Optical photograph of pizeocomposite energy harvester (a). The output voltages of the energy harvester under different loading frequency with same compress pressure of 2.2 MPa (b). The output voltages of the energy harvester under different compressive pressure with same loading frequency of 30 Hz. Its sensitivity is ~13.6 V/MPa and R-squared is 0.978 (c). Voltage responses during 28000 cycles under the compressive pressure of 1.8 MPa (d). Short-circuit current signals of the energy harvester under different compressive pressure with same loading frequency of 30 Hz (e). Output current depend on the load impedance of 2.2 MPa compressive pressure with a frequency of 30 Hz (f).

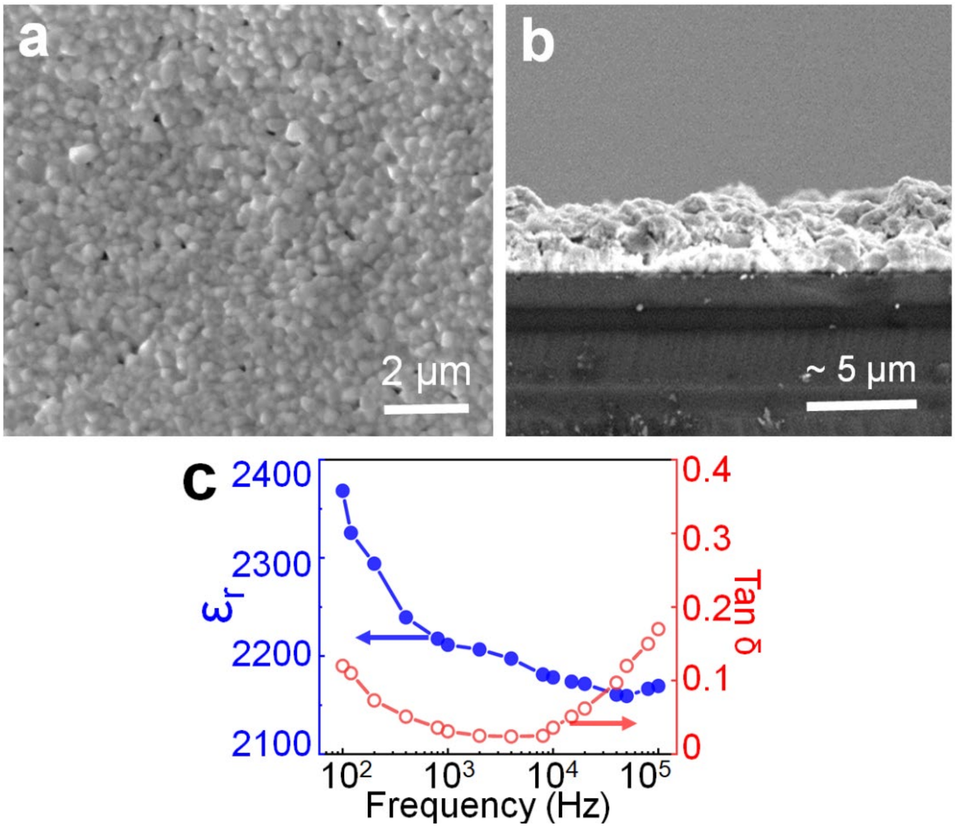

Fig. S16. SEM images of surface topography (a) and cross-sectional topography (b) of the BT films. Frequency dependence of relative dielectric constant ε_r and dielectric loss tan δ for the BT films at 30 °C (c).

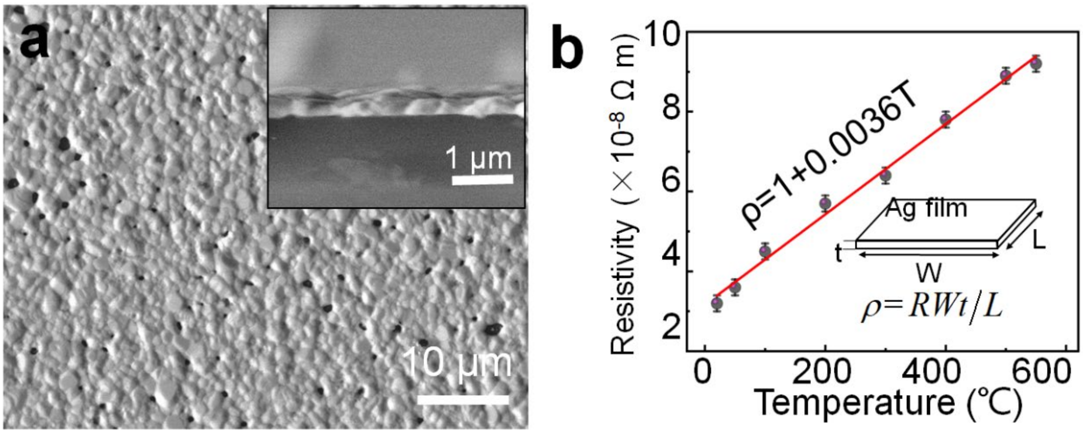

Fig. S17. SEM images of surface topography and cross-sectional topography of the silver films (a). Temperature dependence of resistivity ρ of the printed silver films (b). Inset show the resistivity calculation formula and the temperature coefficient of resistivity (a=0.0036) of the silver films.

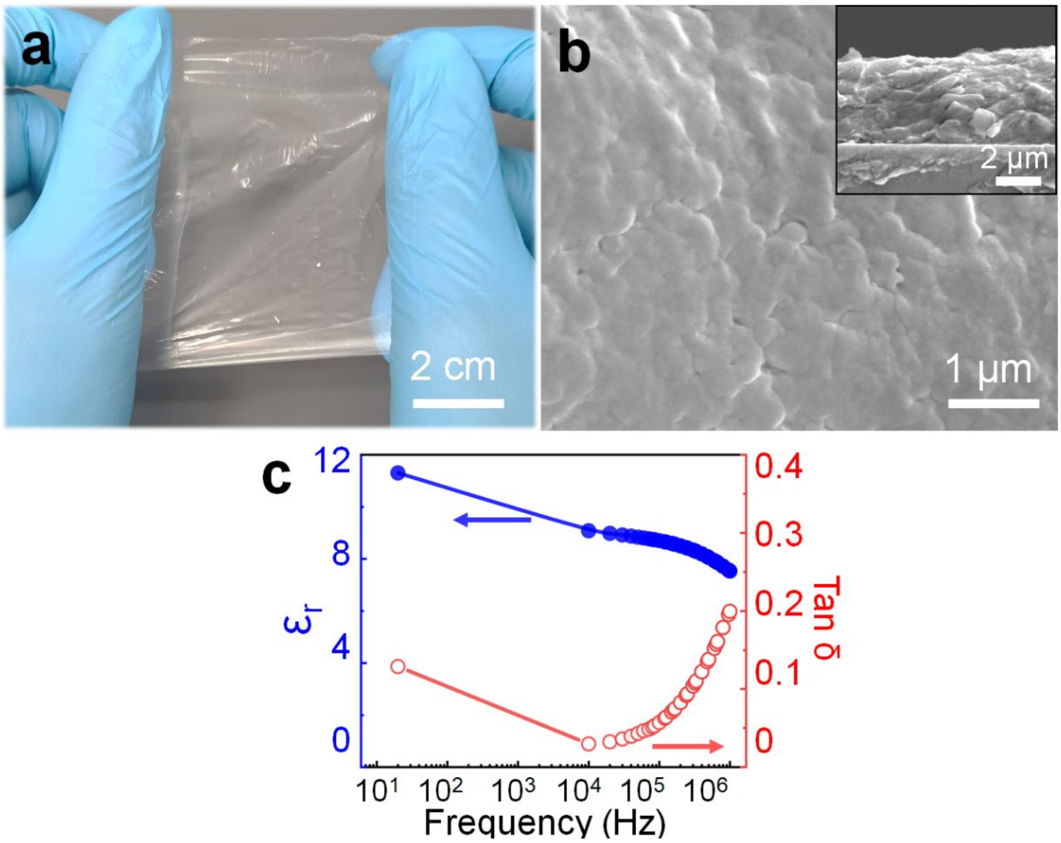

Fig. S18. Photograph showing the PVDF film (a). SEM images showing the surface and cross-sectional topography of PVDF film (b). Frequency dependence of relative dielectric constant ε_r and dielectric loss tan δ for the PVDF films at 30 °C (c).

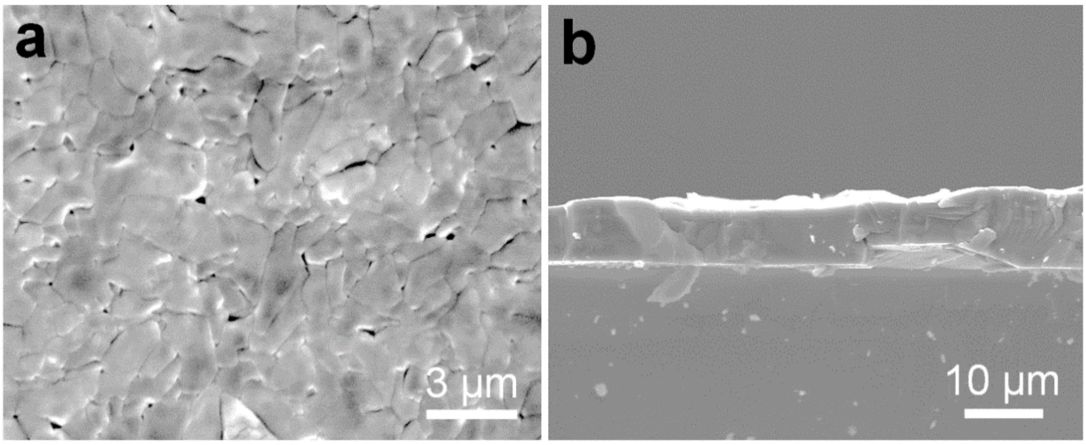

Fig. S19. SEM images of surface topography (a) and cross-sectional topography (b) of the glycine films.

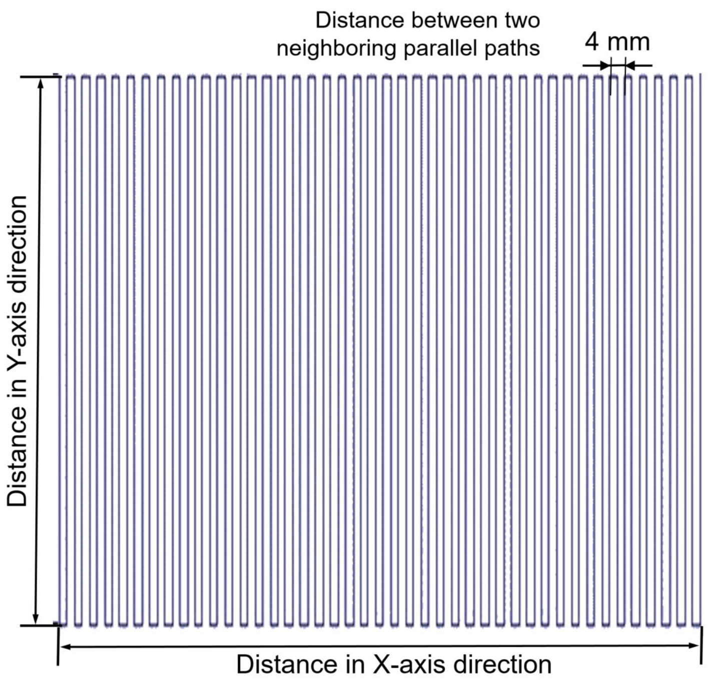

Fig. S20. Schematic demonstration of the X-Y translational stage movement paths for building the films and patterns.

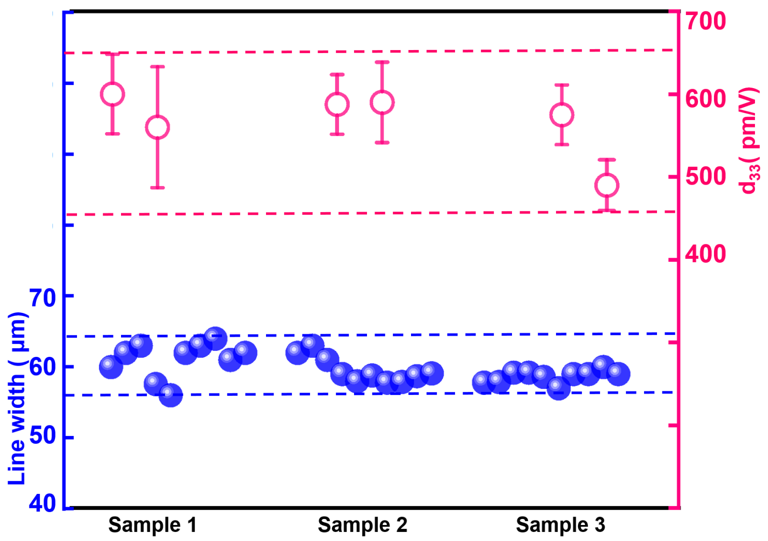

Fig. S21. Distribution of measured line widths for 3 different samples at ten positions (blue dots). No significant differences exist between the width distributions of any of the different positions. Distribution of measured d_{33} for 3 different samples at two positions (pink dots). The tops and bottoms of the dashed line represent the maximum measured value and the minimum measured value, respectively.

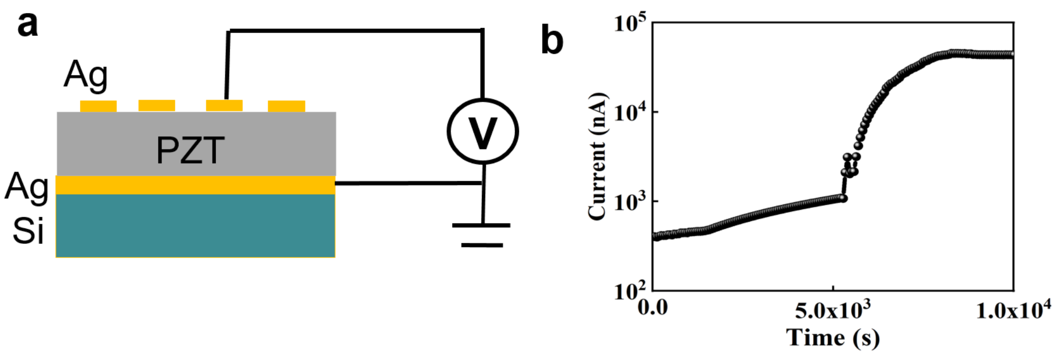

Figure S22. Schematic diagram of circuit used for electrical degradation measurement (a). Variation in leakage current with time for EDP PZT films (b). The measurement is conducted at 180 °C with a DC field of 150 kV/cm.

Supplementary Tables

Table S1. Physical properties of PZT inks for films and patterns fabrication.

**Viscosity (10^{-3} Pas)**	**Surface tension (10^{-3} Nm^{-1})**	**Relative permittivity**	**Electrical conductivity (10^{-3} Sm^{-1})**
1.40	22.9	9.5	6.5

Experimental physical properties of PZT inks are measured at 25 ℃. The viscosity of the PZT slurry is measured using an Ubbelohde viscometer. The surface tension is measured by a contact angle meter (Krüss DSA 100, Krüss GMBH). The relative permittivity is obtained by a precision impedance analyser (4294A, Agilent Technologies).The electrical conductivity is measured by a conductive meter (DDS-307, Shanghai INESA Scientific Instrument). The material properties suitable for obtaining a stable cone-jet mode used in electrohydrodynamic tip streaming process require an electrical conductivity of more than 10^{-11} S m^{-1}, a surface tension of less than 50 mN m^{-1} and a viscosity of less than 100 mPa s^{-1} [4]. It can be seen from Table S1 that our PZT inks meet the requirements for obtaining a stable cone-jet mode.

Table S2. Optimized process parameters for EDP depositing PZT films/patterns.

**Parameter**	**Value**
Ink concentration (mass ratio of particles to sol)	30-50 wt.%
Depositing speed (mm s^{-1})	5~30
Distance between disc to substrate (mm)	2~8
Supply rate of ink (μl min^{-1})	3-200
Applied voltage (kV)	3.0~10.0

Table S3. List of depositing speeds of representative manufacturing techniques for piezoelectric films, which are plotted in Fig. 2h.

**Materials**	**Preparation method**	**Substrates**	**Thickness (μm)**	**Depositing speeds （$\mu m^3\ s^{-1}$）**
PZT films	Pulsed laser deposition[5]	$Pt/Ti/SiO_2/Si$	0.5-5	4.6×10^3-5.4×10^3
PZT films	Magnetron sputtering[6]	$Ti/Pt/SiO_2/Si$	1-2	~2.4×10^7
PZT films	Magnetron sputtering[7]	Si	2-8	5.2×10^7-6.3×10^7
PZT films	Sol-gel[8]	Mica	~2	1.3×10^6-4.9×10^6
PZT films	Sol-gel[9]	Pt/Si	0.97-1.87	1.5×10^6-2.5×10^6
PZT films	Composite sol-gel[8]	Mica	~2	~2.5×10^7
PZT films	Composite sol-gel[10]		16-45	4.2×10^7-1.1×10^8
PZT films	Composite sol-gel[11]	$Pt/Ti/SiO_2/Si$	1	9.3×10^6
PZT films	Aerosol deposition[12]	$Pt/Ti/SiO_2/Si$, Ag-Pd/sapphire	10	5×10^6
BTO films	Aerosol deposition[13]	Steel	1-30	1.6×10^8
PZT-Nb films	Electrophoretic Deposition[14]	Au/Al_2O_3	<20	6.4×10^6-9.6×10^6
PZT films	Electrophoretic Deposition[15]	Platinum	10-100	2.6×10^7-6.5×10^7
**PZT films**	**This work**	**Ag/Si, Ag/mica**	**~4-50**	**2.6×10^8-6.5×10^8**

Table S4. Optimized process parameters for EDP depositing PZT nanoparticles.

Parameter	Value
Sol concentration (M)	0.1-0.4
Substrate temperature (°C)	600
Distance between disc to substrate (mm)	40-80
Supply rate of ink (μl min^{-1})	1-10
Applied voltage (kV)	6.0~10.0

Table S5. Lattice parameters of the EDP deposited PZT films and the dip-coated PZT films, at room temperature.

**Film Type**	**Phase fraction (%)**		**Lattice parameter**				
	Tetragonal	Rhombohedral	a_R (Å)	α_R (°)	a_T (Å)	c_T (Å)	c/a
With electric field	50.336	49.664	4.082	89.649	4.036	4.124	1.0218
Without electric field	49.257	50.743	4.077	89.758	4.040	4.117	1.0192

Table S6. List of piezoelectric properties of representative piezoelectric films fabricated with different manufacturing techniques, which are plotted in Fig. 4h. Pulsed laser deposition (PLD); Aerosol deposition (AD); Laser interferometric vibrometer (LIV); Double beam laser interferometer (DBLI); Pneumatic loading method (PLM); Piezoresponse force microscopy (PFM); low temperature cofired ceramics (LTCC).

Materials	Preparation method	Substrates	Thickness (μm)	d_{33} measurement method or device	d_{33}
PZT(52/48) (001) orientation	PLD[5]	$Pt/Ti/SiO_2/Si$	1-5	DBLI	190-405
PZT(52/48) columnar grains	PLD[16]	$LaNiO_3$/ CNOns/glass	1-5	DBLI	265-458
(T)PZT(30/70)-(R)PZT(55/45) bilayers	PLD[17]	$La_{0.67}Sr_{0.33}MnO_3$/ $SrTiOPLD_3$	0.1	PFM	350
PZT(52/48)	Sputtering[6]	$Ti/Pt/SiO_2/Si$	1	DBLI	160
PZT(40/60)	Sputtering[18]	Nb/SrTiO3	0.2	PFM	135-165
PZT	Sputtering[7]	Si	2-8	DBLI	102-108
PZT(52/48)	Sol-gel[19]	ITO/glass	~2	DBLI	104
PZT(52/48)	Sol-gel[20]	MgO; Al2O3	2	LIV	90-210
PZT	Sol-gel[8]	Mica	~2	LIV	130
PZT(52/48)	Sol-gel[9]	Pt/Si	~1.8	DBLI	75
PZT(52/48)	AD[12]	$Pt/Ti/SiO_2/Si$	10	Berlincourt d_{33} meter	150
PZT-PVDF	AD[21]	Pt/Si	15-80	LIV	115-210
PZT	AD[22]	Pt/ Si	20	LIV	70
PZT(52/48)	Screen printing[23]	$Pt/TiO_2/YSZ/SiO_2/Si$	~35	PFM	184, 339
PZT-PMN	Screen printing[24]	Si; LTCC	80-100	Capacitive detector	140-210
**PZT(52/48)**	**This work**	**Ag/Si, Ag/mica**	**5~20**	**PFM**	**385~590**

References

1. Rosell-Llompart, J. & Fernández de la Mora, J. Generation of monodisperse droplets 0.3 to 4 μm in diameter from electrified cone-jets of highly conducting and viscous liquids. *J. Aerosol Sci.* **25**, 1093–1119 (1994).
2. Hartman, R. P. A., Brunner, D. J., Camelot, D. M. A., Marijnissen, J. C. M. & Scarlett, B. JET BREAK-UP IN ELECTROHYDRODYNAMIC ATOMIZATION IN THE CONE-JET MODE. *J. Aerosol Sci.* **31**, 65–95 (2000).
3. Gañan-Calvo, A. M. 20.O.05 The size and charge of droplets in the electrospraying of polar liquids in cone-jet mode, and the minimum droplet size. *J. Aerosol Sci.* **25**, 309–310 (1994).
4. Smith, D. P. H. The Electrohydrodynamic Atomization of Liquids. *IEEE Trans. Ind. Appl.* **IA-22**, 527–535 (1986).
5. Nguyen, M. D., Houwman, E. P., Dekkers, M. & Rijnders, G. Strongly Enhanced Piezoelectric Response in Lead Zirconate Titanate Films with Vertically Aligned Columnar Grains. *ACS Appl. Mater. Interfaces* **9**, 9849–9861 (2017).
6. Kaden, D. *et al.* Influence of Platinum Bottom Electrodes on the Piezoelectric Performance of PZT Thin Films Hot Sputtered in a High Volume Production Tool. *MRS Proc.* **1397**, mrsf11-1397-p13-31 (2012).
7. Dudde, R., Kaden, D., Quenzer, H. J. & Wagner, B. Preparation of functional PZT films on 6''and 8''silicon wafers by high rate sputtering. *Proc. Nanotech* (2010).
8. Liu, S., Zou, D., Yu, X., Wang, Z. & Yang, Z. Transfer-Free PZT Thin Films for Flexible Nanogenerators Derived from a Single-Step Modified Sol–Gel Process on 2D Mica. *ACS Appl. Mater. Interfaces* **12**, 54991–54999 (2020).
9. Moriyama, M., Totsu, K. & Tanaka, S. Sol–gel deposition and characterization of lead zirconate titanate thin film using different commercial sols. *Sensors Mater.* **31**, 2497–2509 (2019).
10. Shakeri, A., Abdizadeh, H. & Golobostanfard, M. R. Synthesis and characterization of thick PZT films via sol–gel dip coating method. *Appl. Surf. Sci.* **314**, 711–719 (2014).
11. Tsai, C.-C. *et al.* Study of Pb(Zr0.52Ti0.48)O3 microelectromechanical system piezoelectric accelerometers for health monitoring of mechanical motors. *J. Am. Ceram. Soc.* **102**, 4056–4066 (2019).
12. Hahn, B.-D. *et al.* Effects of Zr/Ti ratio and post-annealing temperature on the electrical properties of lead zirconate titanate (PZT) thick films fabricated by aerosol deposition. *J. Mater. Res.* **23**, 226–235 (2008).
13. Khansur, N. H. *et al.* Room temperature deposition of functional ceramic films on low-cost metal substrate. *Ceram. Int.* **44**, 16295–16301 (2018).
14. Bernardo, M. S., Malič, B. & Kuscer, D. PZT-Based Thick Films Prepared by Electrophoretic Deposition from Suspensions with Different Alcohol-Based Solvents. *J. Electrochem. Soc.* **162**, D3040–D3048 (2015).
15. Ngo, T. N. M. *et al.* Fabrication of PZT Thick Film by Electrophoretic Deposition on the Platinum Substrate. *Can Tho Univ. J. Sci.* **14**, 43–53 (2022).
16. Nguyen, M. D., Houwman, E. P. & Rijnders, G. Large piezoelectric strain with ultra-low strain hysteresis in highly c-axis oriented Pb(Zr0.52Ti0.48)O3 films with columnar growth on amorphous glass substrates. *Sci. Rep.* **7**, 12915 (2017).
17. Huang, H.-H. *et al.* Epitaxial PbZrxTi1−xO3 Ferroelectric Bilayers with Giant

Electromechanical Properties. *Adv. Mater. Interfaces* **2**, 1500075 (2015).

18. Bühlmann, S. Electrical Nanoscale Training of Piezoelectric Response Leading to Theoretically Predicted Ferroelastic Domain Contributions in PZT Thin Films. *Adv. Mater.* **20**, 3090 (2008).
19. Hua, H., Chen, Y., Tao, Y., Qi, D. & Li, Y. A highly transparent haptic device with an extremely low driving voltage based on piezoelectric PZT films on glass. *Sensors Actuators A Phys.* **335**, 113396 (2022).
20. Jeong, C. K. *et al.* Flexible highly-effective energy harvester via crystallographic and computational control of nanointerfacial morphotropic piezoelectric thin film. *Nano Res.* **10**, 437–455 (2017).
21. Han, G. *et al.* Effect of Film Thickness on the Piezoelectric Properties of Lead Zirconate Titanate Thick Films Fabricated by Aerosol Deposition. *J. Am. Ceram. Soc.* **94**, 1509–1513 (2011).
22. Hahn, B.-D. *et al.* Fabrication of lead zirconate titanate thick films using a powder containing organic residue. *Jpn. J. Appl. Phys.* **47**, 5545 (2008).
23. Kim, Y.-B., Kim, H.-J., Cheon, C. Il, Choi, D.-J. & Kim, T. S. Electrical properties of 3-component piezoelectric thick films by screen printing method. *Integr. Ferroelectr.* **41**, 107–117 (2001).
24. Gebhardt, S., Schönecker, A. & Bruchmann, C. Integrated Actuators Based on PZT Thick Films for Microsystems Applications. in *Proc. ACTUATOR* 122–125 (2010).
