# Supplementary material for: Fast and versatile electrostatic disc microprinting for piezoelectric elements
Source: Nat Commun. 2023 Oct 14;14:6488. doi: 10.1038/s41467-023-42159-9 (PMC10576804; doi:10.1038/s41467-023-42159-9)
Supplement: Supplementary file 3 — Description of Additional Supplementary Files [file 41467_2023_42159_MOESM3_ESM.pdf]

Description of Additional Supplementary File

Movie Legends

Supplementary Movie 1: Electrostatic disc microprinting process showing the formation of multi-jets.

Supplementary Movie 2: 12 LEDs lit up by the HIPCT device through palm tapping.
